# Play Behaviors in Children during the COVID-19 Pandemic: A Review of the Literature

**DOI:** 10.3390/children8080706

**Published:** 2021-08-17

**Authors:** Anastasia Kourti, Androniki Stavridou, Eleni Panagouli, Theodora Psaltopoulou, Maria Tsolia, Theodoros N. Sergentanis, Artemis Tsitsika

**Affiliations:** 1 2nd Department of Pediatrics, “P. & A. Kyriakou” Children’s Hospital, School of Medicine, National and Kapodistrian University of Athens, 115 27 Athens, Greece; anastasiakourti.ak@gmail.com (A.K.); stavroniki@hotmail.com (A.S.); eleni72000@yahoo.gr (E.P.); mtsolia@med.uoa.gr (M.T.); tsergentanis@yahoo.gr (T.N.S.); 2 Department of Clinical Therapeutics, “Alexandra” Hospital, School of Medicine, National and Kapodistrian University of Athens, 115 28 Athens, Greece; tpsaltop@med.uoa.gr

**Keywords:** play, children, COVID-19, wellbeing, psychosocial development

## Abstract

Play is a key factor for children’s healthy psychological, emotional, social, and cognitive development. During the COVID-19 pandemic, it has been postulated that children’s play was affected, not only regarding the time children spent playing but also in terms of the qualitative characteristics of play. The aim of this review was to investigate how children’s play has changed during the COVID-19 pandemic. A review was conducted in the PubMed, Google Scholar, EMBASE, SCOPUS, ERIC, PsycInfo, and JSTOR databases up to 6 December 2020. Furthermore, references of eligible studies as well as of relevant articles were searched using a snowballing technique. The search retrieved 17 eligible studies, conducted in Europe and North America. In general, outdoor play was reduced during the pandemic; on the other hand, there was an increase in indoor play and in videogames-screen time. COVID-19 was present in children’s pretend play. Children’s play was a key contributor to children’s mood and wellbeing. Furthermore, teachers were especially concerned about how children’s play was affected during the lockdown measures. There is evidence that children’s play habits were affected during the COVID-19 pandemic; further research is required, especially cross-culturally oriented.

## 1. Introduction

Play is a constitutional right for children, in accordance with the Convention on the Rights of the Child (Article 31), which highlights the “right to rest and leisure, to engage in play and recreational activities appropriate to the age of the child and to participate freely in cultural life and the arts” [1]. According to Eberle [2], “play is a roomy subject, broad in human experience, rich and various over time and place, and accommodating pursuits as diverse as peekaboo and party banter, sandlot baseball and contract bridge, scuba diving and Scrabble”. In fact, play is considered a universal, intrinsic activity in children, present in every civilization, despite cultural differences [3]. The importance of play in children’s social, emotional, and mental health has been argued by many specialists.

Play can be divided into three stages: embodiment, projection, and role play, collectively referred to as EPR [4]. The first one includes activities that are experienced through body (e.g., messy play, hopping, jumping); in the second stage, children use different media to express themselves (e.g., paper, colors, clay), while in the latter stage, children develop characters and stories through verbal and non-verbal means. Usually, the three stages of EPR are completed by the age of 7 years [5]. Different types of play could also be used regarding the means and manners (e.g., constructive play, fantasy play [5]), allowing children to explore the world, develop their skills, and enhance resilience. Moreover, through play, children can learn how to handle their fears and adopt adult behaviors as they grow older, while play is very beneficial for children’s physical and cognitive development [6,7,8,9,10,11,12,13]. Child–caregiver reciprocal interaction is mediated through play, offering children a healthy emotional and cognitive development [14].

Regarding the relationships that are formed during play engagement, six stages have been identified [15]: (i) unoccupied play: children do not play but they occupy themselves watching anything that seems exciting to them. Otherwise, they play with their own body, follow their teacher, or just stand around; (ii) onlooker play: in this stage, children observe other children’s play; (iii) solitary play: children play on their own even if they are in a room full of other children, without even noticing them; (iv) parallel play: children in this stage play next to each other without playing together, sharing toys or activities; (v) associative play: children play the same game, but they do not form connections with each other; and (vi) cooperative play: children that have reached the cooperative play stage are able to play with their friends while learning social skills.

Along with the changes in children’s everyday life during the COVID-19 outbreak, it has been postulated that play has also changed. Concerns about the fewer opportunities for learning and play for children due to school closures, home confinement, social distancing, and lack of or limited access to outdoor activities have been reported [16,17]. Children’s pretend play might also have changed, with COVID-19 invading their everyday play; for instance, children might pretend to be doctors and give medication due to COVID-19, as suggested in anecdotal evidence [18]. In many countries, governments have published guidelines regarding children’s healthy play behaviors during the COVID-19 pandemic [19].

This review aims to examine how children’s play has been affected during the COVID-19 pandemic, both quantitively and qualitatively, and how these changes have affected children’s everyday life and wellbeing. Any differences related to gender, age, or other sociodemographic characteristics were explored.

## 2. Methods

### 2.1. Selection of Studies

Relevant studies were sought in the following databases up to 6 December 2020, concerning articles published in 2020 (time of the COVID-19 pandemic): PubMed, Google Scholar, EMBASE, SCOPUS, ERIC, PsycInfo, and JSTOR. Search terms included “outdoor play”, “pretend play”, “symbolic play”, “imaginary play”, “imaginative play”, “play behavior”, “video games”, “outdoor activities”, “sports”, “childhood”, “child”, “children”, “kid”, “kids”, “COVID-19”, “SARS-CoV-19”, “SARS-CoV-2”, “2019-nCoV”, and “novel coronavirus”. In order to include as many related studies, references of eligible studies as well as of relevant articles were searched using a snowballing technique.

Articles that examined play behaviors and play in general in children during the COVID-19 outbreak were considered eligible. Considering play, indoor activities such as videogaming, playing board games, role playing, etc. and outdoor activities such as biking, free play, sports, etc. were eligible. As far as study design is concerned, case reports, cohort studies, cross-sectional studies, case series, and case-control studies were included. Studies evaluating reports from parents were also deemed eligible, in order to describe a comprehensive picture of children’s behaviors. Studies written in English, Spanish, and French were chosen, and there was no gender restriction.

Two authors (A.K. and A.S.) working independently from one another in pairs performed the selection of studies. Data from eligible studies were extracted, including name of first author, region/country where the survey was conducted, language, study period, study design, sample size, age range, selection of sample, ascertainment and/or association with the COVID-19 epidemic, outcomes and methods/questionnaires used for measurements, statistical analysis, and main findings.

### 2.2. Quality Assessment

The Newcastle Ottawa Scale for Cross-sectional Studies [20] and Newcastle Ottawa Scale for Cohort Studies [21] (Appendix A, Table A1 and Table A2) were used to evaluate the risk of bias in eligible studies. The quality assessment was performed by two independent researchers (A.K. and A.S.).

## 3. Results

### 3.1. Selection of Studies

Seventeen studies on the topic were identified. Among them, six studies were conducted in America (four in Canada [22,23,24,25], one in the USA [26], and one in Mexico [27]) and 11 were performed in Europe (five in Spain [28,29,30,31,32], two in Portugal [33,34], two in Ireland [35,36], one in the UK [37], and one in Poland [38]). Four of them had overlapping samples, but they have been included in Table 1 in the “overlapping studies” section” as they offered additional information regarding the issue examined in this paper. The majority of studies were cross-sectional (*n* = 14), and only three were cohort studies (Table 1). A total of 10,313 parents with one or more children 0–17 years old, 2159 children 0–13 years old who were represented by their parents (not specified if one or both parents participated in the research or how many children each parent had), 726 children and adolescents who reported for themselves, and 307 primary teachers reporting on children’s play behaviors during the COVID-19 pandemic were gathered (Table 1. The data of cases where parents reported for their children and specific demographic data for their children were provided (e.g., sex percentage, mean age) are included in Table 1.

### 3.2. Outdoor and Indoor Play

Children’s outdoor play was affected during the COVID-19 pandemic restrictions, with outdoor activities being restricted in regions with more severe measures compared to areas with looser confinement measures [22,30,34]. More specifically, a Canadian study revealed that children and adolescents (5–11 years) only reduced their time spent walking or biking by 47.3%, whereas only 27.7% increase it [23]. In addition, 63.8% of Canadian children participating in the aforementioned study decreased their outdoor physical activity or their engagement with outdoor sports and 47.5% reduced their time in outdoor playing. By contrast, an increase of 31.2% in indoor physical activity or engagement with indoor sports, and an increase of 61.0% in time spent playing inside were noted [23]. On the other hand, 80% of children in an Irish study went for a walk near their neighborhood at least once a week, while 1/3 walked on a daily basis [36].

Primary school children were more likely to spend time outdoors instead of using screens as leisure time (expect during the morning hours due to mandatory attendance of online classes) than their secondary school counterparts [37]. During lockdown, the majority of children aged 5–8 years (97.4%) and 9–13 years (80.6%) preferred free play and/or unstructured physical activity (e.g., running around or other active games) [26].

Decline in time spent on both outdoor and indoor physical activity (walking and biking) and both indoor and outdoor play in children were inversely associated with parental age [24]. Thus, children of older parents spent less time playing. This decline was also positively associated with parents’ encouragement toward physical activity [24], with parents’ support and participation, with household type (detached or not)—with the exception of indoor play [24,33]—and with there being at least one adult free from work [33]. Physical activity was also positively associated with the existence of more than one child in the house and negatively associated with parents working from home [33].

A study on Spanish children revealed that 40.1% practiced some kind of physical activity at home, including dancing and sports, almost every day, 22.2% various times a day, whereas 37% of them never practiced any kind of physical activity or did so only a few times per week [29]. On the other hand, reading books and stories was a more unpopular option for children (25.6% did not read any books, 32.6% read only a few times, and 29.7% read books almost every day, while only 12.1% did so multiple times a day), although sufficient access to books and board games at home was reported [29].

During the lockdown measures, children’s free play (under 12 years old) was negatively associated with the number of television and videogame devices at home and with time spent on smartphones, tablets, or videogame devices per day [31]. Children in Spain watched television for 80.38 min per day, played videogames for 18.78 min per day, used smart phones for 14.58 min per day and tablets 28.47 min per day [31]. Furthermore, children under 5 years old watched television for 65.33 min per day, used tablets for 17.10 min per day and mobile phones for 8.34 min per day [32]. Both mothers and fathers in the UK spent twice as much time on active childcare during the COVID-19 pandemic (including playing games) versus 2014–2015 (35), while indoor play was positively associated with parental discouragement of children using screens [24].

### 3.3. The Types of Play

During lockdown, a Mexican study examined the changes in children’s play behaviors during the COVID-19 pandemic [27]; according to their parents’ observations, 21.7% of children were more engaged with active games, 18.4% with board games, 16.4% with videogames, and 11.7% preferred manipulative games with toys (games using objects to explore a concrete idea), while an increase in violent games compared to before COVID-19 was noted (10.5%). Moreover, 9.4% of parents reported that their children liked role playing, 8.6% played creative types of games, 8.2% engaged in various recreational activities, and 5.7% in exploratory games. The majority of these were fantasy games (49.5%), while 1.1% were skills games and 8% educational games, with 19.5% of them representing the current situation (i.e., pandemic-focused games) and 18.5% of them being games focusing on helping others or showing solidarity in general. More than one third of children (35.6%) preferred to play with adults and 35.2% alone, while only 28.9% preferred to play with other children [27].

The emotional state of children was affected by the lack of social play during COVID-19 lockdown; the majority missed their friends (90%) or playing with other children in general (87%), while approximately one third of them (34%) included COVID-19 as part of their play [36]. Playing with family members was associated with stronger bonds within the family and improvement in children’s mood [38]. In fact, according to Martinez et al. [29], playing (both videogaming and conventional play) was the second most important factor that contributed to children’s happiness (right after family). Furthermore, screen time included not only videogames or social media roaming [30,34], but also different types of physical activity (e.g., team sports, yoga, martial arts, gym sessions), offered through streaming services (in countries such as the USA, where this feature was offered), with older children (9–13) engaging five times more frequently in remote team sports activities than their younger counterparts (OR = 5.40, 95% CI 1.70–17.15) [26].

Parents were also concerned about their children’s play behaviors. More specifically, parents who needed childcare for their children experienced anxiety regarding hands-on play and screen time [25], while a Spanish study showed that having parents of foreign origin was negatively associated with physical activity time [30].

Teachers’ role in enhancing play among children was important as well [35]. The majority of teachers encouraged parents to engage in playful activities during lockdown. During online learning, 68.8% of them used educational games and other activities, while 87% were eager to use play as a mediator in face-to-face learning after returning to the classroom.

### 3.4. Risk of Bias

Of 14 cross-sectional studies, 1 scored 0/10, 2 scored 4/10, 1 scored 5/10, 3 scored 6/10, 6 scored 7/10, and 1 scored 8/10 in the Newcastle–Ottawa Scale. In most studies (*n* = 10), the tool for accessing children’s play was not validated (Table A1). Regarding cohort studies, all three of them were of good quality; however, the non-responder rate was not justified (Table A2).

## 4. Discussion

The COVID-19 pandemic created a new reality, affecting all members of the global community, including children, as one of the most vulnerable groups. Those changes were reflected not only upon access to education and physical and psychological safety, but also upon social and physical activities [39,40]. This review addressed an important factor in children’s life during the ongoing COVID-19 pandemic, namely, children’s play behaviors, providing valuable data regarding both children’s indoor and outdoor activities. Various data from different regions of Europe and North America were retrieved, including 17 articles [22,23,24,25,26,27,28,29,30,31,32,33,34,35,36,37,38].

According to this review, a decrease in play behaviors was noted in outdoor activities, due to confinement measures across countries [22,29,30,33]. Similar findings were provided by Graber et al. describing that children’s access to play was limited during periods of quarantine (e.g., hospitalization, refugee camp), but not providing a significant change in children’s play behaviors overall [41]. Indeed, most studies concluded that children decreased the time spent on physical activity and mostly on outdoor activities. In most countries, restrictive measures prohibited or at least discouraged parents from using playgrounds or outdoor sports; thus, outdoor activities were limited among both boys and girls [23]. Those findings represent a challenge in light of the guidelines of the World Health Organization, which defines that children and adolescents aged 5–17 years old should exercise, in moderate to high intensity, at least 60 min daily, mainly through aerobic exercises [42]. Overall, outdoor play connects children with nature and make them more active and curious, while also boosting their immune system and regulating their sleep routines [43,44].

During lockdown, children spent their time on various indoor activities. According to this review, children often chose videogames using a television, PC, tablet, or smartphone, while reading books and playing board games were not so popular [29,31]. Studies during the ongoing COVID-19 pandemic noted that there was an increase in screen time among children, not only for educational purposes, but also for leisure activities [45]. The American Pediatric Association proposed that children’s time in front of screens should be determined by their age, providing parents with a useful guide [46].

Although there were limitations in activities, both indoor and outdoor, children never gave up their imagination. They chose different types of play activities, including their siblings or parents in them whenever they were available, leading to stronger bonds and improving their mood [27,28,34,38]. Additionally, the importance of play among children emerged as a stress coping mechanism [40]. In general, children engaging in play and related activities also present with better social skills, enhanced cognitive function, reduced anxiety, and fewer depressive symptoms [47,48]. It is, however, important to underline the fact that social play has been limited during the pandemic, and children shared that they missed their friends [36]. Since the COVID-19 outbreak and the measures taken by each country is an unprecedented situation, we do not know yet the impact that it may have on children’s and adolescents’ peer bonding, or whether children and adolescents have discovered other means and coping mechanisms (e.g., video calls with their friends).

Commenting on the external validity of this review, all studies were conducted in countries with high-income economies—with the exception of the study by Torres Gonzalez et al. [27], which was conducted in an upper-middle-income country (Mexico); studies were performed in Europe and America; there were no eligible studies from countries with a different socioeconomic background and culture (e.g., from African or Asian countries). Moreover, a lack of investigation around associations with the sociocultural background of the families was noted.

Play activities are an integral part of children’s everyday life, incorporating physical, mental, and psychosocial benefits. The importance of maintaining a play routine is essential, but the new reality imposed by the ongoing COVID-19 pandemic has disrupted it. Parents, in cooperation with teachers and health providers, should reinforce children’s opportunities to engage in both outdoor and indoor activities, during and after the ongoing COVID-19 pandemic.

## Figures and Tables

**Table 1:** Descriptive characteristics of included studies.

First Author (Year)	Region, Country	Language	Study Period	Study Design	Sample	Sample Size	Age Range	Selection of Sample	Outcomes, Way/Questionnaires They Were Measured	Main Findings
Andrew et al. [37] (2020)	UK	English	29 April to 20 June 2020	Cross-sectional	Parents with at least one child between the ages of 2 and 15	5.582	NR	Nationally representative 2019 Labour Force Survey (LFS)	Interview	The rest of the children’s days are filled with learning and leisure activities, which include playing, reading, being outdoors, socializing and on-screen time. Comparing primary with secondary school children in the lockdown period, we see that primary children are more likely to spend time outdoors in a given hour and less likely to be on-screen except for the early hours of the morning.
Arufe Giráldez; Cachón Zagalaz et al. [31] (2020)	Spain	Spanish	23 March 2020–6 May 2020	Cross-sectional	Parents of children in Spain	837 (50.2% males)	<12 years old (M = 6.22) 0–2 years old: 202, 3–6 years old: 260, 7–12 years old: 375	Through social networks and using as a filter families with residence in Spain and children under 12 years old	Equipamiento y Uso de Tecnologías de Información y Comunicación en los Hogares (TIC-H2019) in Spanish, self-registration sheet ad hoc questionnaire for parents	Only 13.1% of children spend adequate time on everyday physical activity. Children that spend less time playing videogames, using a computer, watching television, and using tablets or mobile phones spend more time on physical activities.
Dunton et al. [26] (2020)	US, 35 states and the District of Columbia.	English	25 April to 16 May 2020 and a second online survey was scheduled to occur within 6–12 months	Cohort	Children	211	5 to 13 years	Respondents were electronically invited through various social media platforms (e.g., Facebook, Twitter) and university-based emailing lists of students, faculty, and staff.	Online self-report questionnaire	90% of children during the early-COVID-19 period chose free play/unstructured activity (e.g., running around, tag) and 55% went for a walk; 10.4% of children participated in team sports training sessions or practice through remote or streaming services, 28.9% participated in activity classes or lessons (e.g., martial arts, dance, yoga) through remote or streaming services, and 2.4% participated in remote or streaming classes or sessions provided by a health club or gym. Older children (age 9–13) vs. younger children (ages 5–8) were more than five times as likely to participate in team sports training session or practice through remote or streaming services (OR = 5.40, 95% CI [1.70, 17.15], Wald = 8.19, *p* = 0.004).
Egan et al. [36] (2021)	Ireland	English	21 May to 3 June 2020	Cross-sectional	Parents of children 1–10 years old	512	1–10	Cognition, Development, and Learning Lab	Interview	90% missed their friends, 87% missed playing with other children, 72% said play was affected by restrictions, and 34% brought the virus into their play. Most children spent more time playing outdoors, with games and toys and on-screen activities, and 80% went for a walk in their neighborhood at least once a week, with over a third going for a walk every day.
Gambin et al. [38] (2020)	Poland	English	4–8 May 2020	Cross-sectional	Parents	459	18–73	Online from the Polish research panel ARIADNA	Brief version of the Empathic Sensitivity Questionnaire, The Difficulties in Emotion Regulation Scale Short Form, Social Support Scale, Parenting Self-Agency Measure, as well as The Scale of Positive Experiences in Parent-Child Relationship during the COVID-19 lockdown	Parents and children may have some positive experiences in their relationship during lockdown. Maybe, in some families, experiencing distress or emotional arousal caused parents to be more sensitive and focused on children and relations with them to provide protection or help, so despite struggling with a higher level of negative emotions, they noticed new achievements from their children, shared the joy of playing with them or felt the satisfaction of creating new activities.
Giménez-Dasí et al. [28] (2020)	Madrid, Spain	English	March 2020, and 8–25 April 2020	cohort	Families with children aged between 3.2 and 11.1 years	167	NR	Children recruited from two public school in Madrid.	System of Evaluation of Children and Adolescents (SENA) and online	Positive changes in some families, referring to improvements in mood and to the positive effect of the greater availability of free time and family time.
Martinez et al. [29] (2020)	Spain	Spanish	21 March 2020–5 April 2020	Cross-sectional	Parents of children and adolescents in Spain	435 (44.0% males, 3.8% not stated)	8–17 years old (8–12, 13–17)	Via social networks and survey on LimeSurvey platform	25-question questionnaire for children (both open and close questions)	9.30% of male participants and 3.70% of female participants (4.30% 8–12 years old, and 10.70% 13–17 years old) did not practice any sport or carry out any physical activity during the confinement measures. 21.50% of male participants and 13.40% of female participants (14.10% 8–12 years old, and 14.10% 13–17 years old) did not practice any hobby during the confinement measures. Furthermore, playing (both online gaming and conventional games) was the second most important factor (after family) for children and adolescents’ happiness (22.77% boys <12 years old, 26.09% girls <12 years old, 16.65 male adolescents (13–17), 25.86% female adolescents (13–17)). 23.77% of the participants liked the fact that they had more free time to play at home.
Medrano et al. [30] (2020)	Navarra (Spain)	English	September–December 2019 and March–April 2020	Cohort	Children and adolescents in Spain	291 children (12.1 ± 2.4 years at baseline, 47.8% girls) included in the MUGI project and 113 children from the whole sample agreed to participate in the second evaluation and completed the online questionnaire (39% participation rate; 12.0 ± 2.6 years at baseline, 48.7% girls).	8 to 16 years	MUGI project	Demographic values, The Youth Activity Profile” questionnaire (YAP), Mediterranean Diet Quality Index for children and teenagers (KIDMED) questionnaire, lifestyle questionnaires during confinement.	During the COVID-19 confinement, physical activity (−91 ± 55 min/d, *p* < 0.001) and screen time (±2.6 h/d, *p* < 0.001) worsened, whereas the KIDMED score improved (0.5 ± 2.2 points, *p* < 0.02). The decrease in PA was higher in children with a mother of non-Spanish origin (−1.8 ± 0.2 vs. −1.5 ± 0.1 h/d, *p* < 0.04) or with non-university studies (−1.7 ± 0.1 vs. −1.3 ± 0.1 h/d, *p* < 0.005) in comparison to their counterparts.
Moore et al. [24] (2020)	Canada	English	1 month after the WHO declared COVID-19 a global pandemic	Cross-sectional	Parents of children and adolescents 5–17 years old	1474 children and adolescents (46% males)	5–17 (5–11 and 11–17)	Survey via Maru/Matchbox	Canadian 24-Hour Movement Guidelines for Children and Youth	1–5 scale (3 no change): Walks or bikes in neighborhood: children overall 2.57, boys 2.54, girls 2.61. Physical activity or sports outside: children overall 2.28, boys 2.26, girls 2.30. Physical activity or sports inside: children overall 2.94, boys 3.01, girls 2.88 Outdoor play: children overall 2.58, boys 2.57, girls 2.59. Indoor Play: children overall 3.85, boys 3.86, girls 3.84.
O’Keeffe, C. and McNally, S. [35] (2020)	Republic of Ireland (ROI)	English	26 June to 10 July 2020	Cross-sectional	Primary school teachers	309	20–69	Qualtrics online platform accessed via a link shared on social media platforms	Online self-report questionnaire (32-item list)	During confinement: (i) there was a decrease in children’s physical activity time (72.3%) and an increase in screen time (71.3%) and family activities (83.9%); (ii) the only sex differences were found in playful screen time (boys > girls) and in play without PA (girls > boys); (iii) among age groups, there was a trend for an increase in overall sedentary time and a complementary decrease in overall physical activity time (both F(3,2097) = 97.951, *p* < 0.001).
Pombo, A., Luz, C., Rodrigues, L.P., Ferreira, C. & Cordovil, R. [33] (2020)	Portugal	English	23 March and 1 April 2020	Cross-sectional	Children younger than 13 years	2159	0–2 years: 462; 3–5 years: 765; 6–9 years: 606; and 10–12 years: 326	Survey on LimeSurvey, hosted on the Faculty of Human Kinetics, University of Lisbon.	Online questionnaire including four factors: 1. Household: 2.Housing characteristics. 3. Household routines 4. Children’s routines.	The majority of teachers reported encouraging parents to play with their children during lockdown, and 68.8% reported the widespread use of play as one of the key methodologies to engage children in online learning during the pandemic. In all, 87% of teachers indicated that play would have a significant role in teachers’ approaches to supporting children’s transition to school upon reopening, but there would be limited capacity for play in the classroom due to COVID-19 regulations.
Torres González et al. [27] (2020)	Mexico	English	21 April to 5 March 2020	Cross-sectional	Parents of children from 1–12 years old under voluntary social isolation by COVID-19	365	Over 18 years old (mean = 35.62, SD = 7.4)	Open invitation through social networks and emailed to the participants	Online self-report questionnaire (35 item list)	21.7% perceived a greater presence of active games, 18.4% perceived an increase in participation in board games, 16.4% in videogames, 11.7% in manipulative games with toys, 9.4% reported roleplay as predominant, 8.6% showed a bigger frequency of engaging in creative types of games, 8.2% different recreational activities, and 5.7% exploratory games. Regarding game themes, 49.5% of the parents mentioned that they observed a greater predominance of fantasy games, with 19.5% of these games representing the current situation, 18.6% representing games of solidarity and mutual help, 10.5% an increase in violent games, 1.1% challenge/skill games, and the remaining 0.8% stating that their children played mostly exploratory/educational games. In addition, parents reported that 35.6% of children prefer to play with adults, 35.2% alone, and 28.9% with other children.
Stienwand, S. et al. [25] (2020)	Canada	English	14 April to 1 June 2020	Cross-sectional	Mothers and fathers with children aged 2–8 years	708	35.59 years old (SD = 5.59; range = 21–72)	Online convenience sample through online advertisements, posters shared on social media platforms, and indirect recruitment through media interviews discussing the pandemic and physical distancing guidelines	Online questionnaire, self-report Adult Mental Health Disorder Checklist (AMHDC), The Center for Epidemiologic Studies Depression (CESD) and Revised (CESD-R), Generalized Anxiety Disorder 7-Item Scale (GAD-7), Parenting Stress Index (PSI), Parenting Strategies During COVID-19.	For families with childcare needs, parental anxiety was associated with higher total hands-on play (F(3142) = 14.01, *p* < 0.001), combined hands-on play (F(285) = 6.82, *p* = 0.011), and combined screen time (F(282) = 6.25, *p* = 0.014). Families without childcare needs indicated parenting stress was associated with lower total hands-on play (F(3212) = 7.95, *p* < 0.005) and combined hands-on play (F(2110) = 5.67, *p* = 0.019), and higher supervised screen time (F(3138) = 6.14, *p* = 0.014).
**Studies with overlapping samples**										
Arufe Giráldez, Sanmiguel-Rodríguez et al. [32] (2020) (overlapping with Arufe Giráldez, Cachón Zagalaz et al. [31] 2020)	Spain	English	23 March 2020–6 May 2020	Cross-sectional	Parents of children in Spain	280 (51.1% males)	0–4 years old (M = 2.44), <0 years old: 9.3% (*n* = 26); 1–2 years old: 40.4% (*n* = 113); 3–4 years old: 50.4% (*n* = 140)	Through social networks and using as a filter families with residence in Spain and children under 5 years old	Equipamiento y Uso de Tecnologías de Información y Comunicación en los Hogares (TIC-H2019) in Spanish, self-reported questionnaire for parents	Overall decrease in outdoor play. Differences in outdoor play across different Canadian provinces.
De Lannoy et al. [22] (2020) (overlapping with Moore et al., [24] 2020 and Mitra et al., [23] 2020	Canada	English	1 month after the WHO declared COVID-19 a global pandemic	Cross-sectional	Parents of children and adolescents 5–17 years old	1472 children and adolescents (46% males)	5–17 (5–11 and 11–17)	Survey via Maru/Matchbox	Canadian 24-H Movement Guidelines for Children and Youth	Walk or bike: all children and youth: decrease 53.2%, same 26,3%, increase 20.5%; children (5–11 years): decrease 47.3%, same 25.0%, increase 27.7%; youth (12–17 years): decrease 58.4%, same 27.6%, increase 14.0%Physical activity or sport outside: all children and youth: decrease 63.8%, same 22.2%, increase 20.5%; children (5–11 years): decrease 47.3%, same 25.0%, increase 14.0%; youth (12–17 years): decrease 68.0%, same 21.8%, increase 10.1%Physical activity or sport inside: all children and youth: decrease 34.0%, same 40.5%, increase 25.5%; children (5–11 years): decrease 27.1%, same 41.7%, increase 31.2%; youth (12–17 years): decrease 40.1%, same 39.4%, increase 20.5%Playing outside: all children and youth: decrease 51.2%, same 30.9%, increase 17.9%; children (5–11 years): decrease 47.5%, same 26.3%, increase 26.3%; youth (12–17 years): decrease 54.6%, same 35.0%, increase 10.4% Playing inside: all children and youth: decrease 7.0%, same 39.9%, increase 53.1%; children (5–11 years): decrease 6.5%, same 32.5%, increase 61.0%; youth (12–17 years): decrease 7.4%, same 46.6%, increase 46.0% Increased outdoor activities: House (ref: Apartment) OR = 2.05, Pr < 0.001 Dwelling density OR = 0.77 Pr = 0.079, Access to parks OR = 0.83 Pr = 0.067 Distance to major road OR = 1.22 Pr = 0.039, Intercept OR = 0.59, Pr = 0.029 “Increased outdoor activities, age 7–11 (ref 12–17): OR = 1.77, Pr < 0.001 Income of $75,000 to $150,000 (ref $35,000 to 75,000): OR = 1.46 Pr = 0.000 House (ref apartment): OR = 1.39, Pr = 0.017 Dwelling density: OR = 0.68, Pr < 0.001 Distance to major road: OR = 1.12, Pr = 0.073 Dwelling density X Access to park: OR = 1.19, Pr = 0.002 Intercept: OR = 0.32, Pr < 0.001
Mitra et al. [23] (2020) (overlapping with Moore et al., [24] 2020 and De Lannoy et al., [22] 2020)	Canada	English	1 month after the WHO declared COVID-19 a global pandemic	Cross-sectional	Parents of children and adolescents 5–17 years old	1473–690 children- (46% males)	5–17 (5–11 and 11–17)	Survey via Maru/Matchbox	Parents’ self-report in 11 movement behaviors: 1) walking or cycling, 2) physical activity or sport outside, 3) physical activity or sport inside, 4) household chores, 5) playing outside, 6) playing inside, 7) screen time, 8) social media use, 9) othernon-screen-based sedentary activities, 10) sleep duration, and 11) sleep quality.	Daily physical activity in minutes: ≤ 1 year 6.54 min; 1–2 years 35.58 min; 3–4 years 33.45 min. Creativity level (10-point scale): ≤ 1 year 5.65; 1–2 years 7.70; 3–4 years 8.09
Pombo, A., A., Luz, C., Rodrigues, L.P. & Cordovil, R. [34] (2021) (overlapping with Pombo, A., Luz, C., Rodrigues, L.P., Ferreira, C., and Cordovil, R. [33] 2020)	Portugal	English	23 March and 1 April 2020	Cross-sectional	Children younger than 13 years	2159	0–2 years: 462; 3–5 years: 765; 6–9 years: 606; and 10–12 years: 326	Survey on LimeSurvey, hosted on the Faculty of Human Kinetics, University of Lisbon.	Questionnaire including demographic values, household characteristics, children’s routines, and five categories: a) intellectual activity (school assignments and online classes), b) playful screen time (games, movies, social networks, Internet, audio and video calls), c) play without PA (reading, drawing, painting, board games, cards, Legos, and so on), d) play with physical activity (hide and seek, jumping, tag, and so on), e) PA (organized PA indoors, PA outdoors, walking the dog).	Boys and girls did not differ in the %PA in any of the age groups; children with an outdoor space and who had other children in the household were significantly more active (*p* < 0.001); children from families with all adults working from home showed lower levels of %PA; and being younger, having a big outdoor space, having other children in the household, and having at least one adult not working from home were significant positive predictors of children’s %PA, explaining 21% of the overall variance.

## Data Availability

Data are included within the article.

## Appendix A

**Table A1:** Newcastle–Ottawa for Cross-sectional studies.

	Selection				Comparability	Outcome		Total
First author (year)	Representativeness of the sample	Sample size	Non-respondents	Ascertainment of exposure	The subjects in different outcome groups are comparable based on the study design or analysis. Confounding factors are controlled.	Assessment of outcome	Statistical test	
Andrew et al. [37] (2020)	*	*	-	*	**	*	-	6/10
	parents recruited via online survey company	5582	No description	Non-validated but tool is described	Family structure, employment, gender	Self-report	no	
Arufe Giráldez, Cachón Zagalaz et al. [31] (2020)	-	-	-	**	**	*	*	6/10
	Non-experimental design for the recruitment of the participants	837	No description	Validated measurement tool	Age, number of televisions, PCs and/or tablets at home	Self-report	yes	
Egan et al. [36] (2021)	-	-	-	-	-	-	-	0/10
	No description	No description	No description	No description	No description	No description	no	
Gambin, M. et al. [38] (2020)	*	-	*	*	**	*	*	7/10
	Parents recruited via online platform	459	From 514, 459 were eligible	Non-validated but tool is described	Gender, residence, education, marital status	Self-report	yes	
Martinez et al. [29] (2020)	*	-	-	*	**	*	-	5/10
	Parents recruited via social networks	435	No description	Non-validated but tool is described	Gender and age of the child	Self-report	no	
Moore et al. [24] (2020)	*****	-	*	*	**	*	*	7/10
	Parents	1472	31/1503	Non-validated but tool is described	Gender and age of the child	Self-report	yes	
O’Keeffe, C. and McNally, S. [35] (2020)	*	-	*	*	**	*	*	7/10
	Snowball sampling	309	From 351, 309 were eligible	Non-validated but tool is described	Gender, age, professional characteristics	Self-report	yes	
Pombo, A., Luz, C., Rodrigues, L.P., Ferreira, C., and Cordovil, R. [33] (2020)	*	*	-	*	**	*	*	7/10
	Parents recruited via online survey	2159	No description	Non-validated but tool is described	Age, gender	Self-report	yes	
Stienwandt, S. et al. [25] (2020)	*	*	-	**	**	*	*	8/10
	Parents recruited through online advertisements, posters on social media platforms, and indirect recruitment through media interviews discussing the pandemic and physical distancing guidelines	708	No description	Online questionnaire, self-report Adult Mental Health Disorder Checklist (AMHDC), The Center for Epidemiologic Studies Depression (CESD) and Revised (CESD-R), Generalized Anxiety Disorder 7-Item Scale (GAD-7), Parenting Stress Index (PSI), Parenting Strategies during COVID-19	Maternal and paternal education level, household employment status during COVID-19, total annual household income, marital status, number of children	Self-report	yes	
Torres González et al. [27] (2020)	*	-	-	*	**	*	*	6/10
	Online recruitment: parents of children from 1 to 12 years old under voluntarysocial isolation by COVID-19	365	No description	Non-validated but tool is described	Gender, marital status, socioeconomic status	Self-report	yes	
**Research with Overlapping Sample**								
								
Arufe Giráldez, Sanmiguel-Rodríguez et al. [32] (2020) (overlapping with Arufe Giráldez, Cachón Zagalaz et al., [31] 2020)	-	-	-	**	*	*	-	4/10
	Non-experimental design for the recruitment of the participants	280	No description	Validated measurement tool	Age	Self-report	no	
De Lannoy et al. [22] (2020) (overlapping with Moore et al., [24] 2020 and Mitra et al. [23], 2020)	*****	-	*	*	-	*	-	
	Parents	1472	31/1503	Non-validated but tool is described	No description	Self-report	no	4/10
Mitra et al. [23] (2020) (overlapping with Moore et al [24]., 2020 and De Lannoy et al. [22], 2020)	*	-	*	*	**	*	*	
	Parents	1472	31/1503	Non-validated but tool is described	Gender and age of the child	Self-report	yes	7/10
Pombo, A., Luz, C., Rodrigues, L.P. & Cordovi, R. l, et al. [34] (2021) (overlapping with Pombo, A., Luz, C., Rodrigues, L.P., Ferreira, C., and Cordovil, R. [33], 2020)	*	*	-	*	**	*	*	7/10
	Parents recruited via online survey	2159	No description	Online questionnaire	Age, gender, type of house, availability of outdoor space	Self-report	yes	

According to Newcastle–Ottawa Scale, each study is rated with none, one or two stars (*) [20,21].

**Table A2:** Newcastle–Ottawa for Cohort Studies.

	**Selection**				**Comparability**	**Outcome**			**Total**
First author (year)	Representativeness of the exposed cohort	Selection of the non-exposed cohort	Ascertainment of exposure	Demonstration that outcome of interest was not present at start of study	Comparability of cohorts on the basis of the design or analysis controlled for confounders	Assessment of outcome	Was follow-up long enough for outcomes to occur	Adequacy of follow-up of cohorts	
Medrano, M. et al. [30] (2020)	*	*	*	*	**	*	*	*	good quality
	Children and adolescents in Spain	Same community	MUGI project	yes	Age, sex, school, origin of mother, BMI	Record linkage, self-report	yes	no different	
Dunton GF, Do B, Wang SD [26] (2020)	*	*	*	*	**	-	*	*	good quality
	Children 5 to 13 years	Same community	Structure interview, self-report	Pre-COVID period (February 20,200 and early-COVID-19 period (April–May 2020)	Parental age, sex, marital status, work status, annual household income, child age, sex, ethnicity, race	Self-report	yes	Complete follow-up	
	*	*	*	*		*	*	*	
Giménez-Dasí, M. et al. [28](2020)	Parents of children 1–10 years old	Same community	Self-report	Pre-confinement (February 2020) and 4–6 weeks during confinement	No comparability	Validated tool	yes	Complete follow-up	good quality

According to Newcastle–Ottawa Scale, each study is rated with none, one or two stars (*) [20,21].
